# The Growing Importance of Three-Dimensional Models and Microphysiological Systems in the Assessment of Mycotoxin Toxicity

**DOI:** 10.3390/toxins15070422

**Published:** 2023-06-29

**Authors:** Veronica Zingales, Maria Rosaria Esposito, Noemi Torriero, Mercedes Taroncher, Elisa Cimetta, María-José Ruiz

**Affiliations:** 1 Laboratory of Toxicology, Faculty of Pharmacy, University of Valencia, Av. Vicent Andrés Estellés s/n, 46100 Valencia, Spain; mercedes.taroncher@uv.es; 2 Department of Industrial Engineering (DII), University of Padua, Via Marzolo 9, 35131 Padova, Italy; mr.esposito@irpcds.org (M.R.E.); noemi.torriero@phd.unipd.it (N.T.); elisa.cimetta@unipd.it (E.C.); 3 Fondazione Istituto di Ricerca Pediatrica Cittá Della Speranza (IRP)—Lab BIAMET, Corso Stati Uniti 4, 35127 Padova, Italy

**Keywords:** alternative methods, 3D culture, spheroids, in vitro toxicology, mycotoxins

## Abstract

Current investigations in the field of toxicology mostly rely on 2D cell cultures and animal models. Although well-accepted, the traditional 2D cell-culture approach has evident drawbacks and is distant from the in vivo microenvironment. To overcome these limitations, increasing efforts have been made in the development of alternative models that can better recapitulate the in vivo architecture of tissues and organs. Even though the use of 3D cultures is gaining popularity, there are still open questions on their robustness and standardization. In this review, we discuss the current spheroid culture and organ-on-a-chip techniques as well as the main conceptual and technical considerations for the correct establishment of such models. For each system, the toxicological functional assays are then discussed, highlighting their major advantages, disadvantages, and limitations. Finally, a focus on the applications of 3D cell culture for mycotoxin toxicity assessments is provided. Given the known difficulties in defining the safety ranges of exposure for regulatory agency policies, we are confident that the application of alternative methods may greatly improve the overall risk assessment.

## 1. Introduction

Since the early 1900s, assessing the effects of toxic chemicals mainly relies on in vitro two-dimensional (2D) cell cultures, which poorly reflect in vivo conditions and are affected by several limitations [1,2]. In 2D cultures, cells lack both the in vivo tissue organization and key biological functions such as cell–cell and cell–matrix interactions, all contributing to decreased differentiation, non-physiological distribution of nutrients and growth factors from the medium, reduced resistance to xenobiotics, and rapid proliferation [3]. Due to their inadequate ability to elucidate complex biological processes, 2D cell-based systems often require additional follow-up experiments with animal models to better assess toxicity. However, animal experiments also show evident limitations: they are costly, require relatively large amounts of test substances and longer experimental times [4], and express and regulate genes and proteins differently from humans, limiting the straightforward translation of information [5,6]. Last but not least, laboratory procedures that often lead to animal suffering and their sacrifice are raising ever-growing concerns among the general public, such that various countries and organizations are strictly controlling animal studies due to ethical reasons [7]. Over the last decades, several alternative in vitro toxicity-testing strategies that better mimic the in vivo cell behavior and provide more predictive results have been developed for evaluating the hazards associated with the exposure to toxic substances. In 2007, the Toxicology in the 21st Century program, or Tox-21c, prompted by the National Research Council, dominated the discussion [8]. Since then, numerous conferences have addressed Tox-21c’s call for a paradigm shift in toxicology, several American agencies formed a coalition to facilitate its implementation, and the U.S. Environmental Protection Agency (EPA) made the novel approach its official toxicity-testing strategy [9,10]. Similarly, the 1986 European Directive 86/609/EEC article 7 encourages the development and validation of alternative techniques that would provide the same level of information as animal experiments [11]. Most recently, animals’ wellbeing and welfare in laboratories have been further regulated by Directive 2010/63/EU [12]. However, although Europe is heavily investing in the 3R principles—reduce, replace, and refine—there is a certain reluctance to fully embrace the alternative methods due to the remaining difficulties in standardization, quality control, and validation [4].

The call for alternative and more predictive methods has led to the development of novel advanced in vitro systems with greater physiological relevance.

## 2. Towards More Predictive Toxicology

It is increasingly recognized that cells grown in a 3D environment more closely resemble in vivo cell functions due to the improved cell–cell and cell–matrix interactions [13,14]. Indeed, the phenotype and physiological behavior of individual cells are strongly dependent on interactions with neighboring cells and proteins of the extracellular matrix (ECM) [15]. Moreover, 3D cultures have well-differentiated cells, show more realistic proliferation rates, reacquire functions lost to monolayers, and are more resistant to xenobiotics treatments, providing a more accurate representation of their effects [3,16]. Finally, 3D cell cultures have longer-term stability, making them an appropriate tool for chronic toxicity studies. Overall, 3D models outperform standard 2D monolayer cultures and provide researchers with tools to better analyze poorly understood phenomena. Despite all these evident advantages, 3D cultures tend to be more expensive and time-consuming, technically challenging, and low in throughput and increase the difficulty of interpreting the data and replicating the experiments.

Simplifying, 3D cultures can be divided in two main groups: spheroids and organoids. Whereas spheroids can be defined as a cluster of differentiated cells that aggregate, exhibiting some tissue-like structure, organoids are multicellular self-assembled constructs that mimic the corresponding in vivo organ in terms of cell types, structure, and function [17]. The lack of components of the in vivo organ, such as vasculature [18], prompted the development and integration of micro-physiological systems (organ-on-a-chip, OoC). These technologies, which combine in vitro models with perfusion and micro-engineered environments, enable the creation of controlled micro-environmental niches and patterned biomolecular signals. Since the first published OoC by Shuler et al. [19], the number of studies on this topic has increased constantly. Although useful to accelerate the preclinical assessment of substances on cells and tissue mimics, they present limitations due to the lack of the complex inter-organ crosstalk in the human body. To improve the available models, researchers have thus devised various “multi-organ-on-a-chip” devices, connecting multiple organs on a single chip via microfluidic channels, enabling interactions through the flow of culture medium [20,21,22,23,24]. Organ-chip models are obviously more difficult to use than other 3D culture techniques in terms of development, sample handling, and manipulation [25].

Given this overview of in vitro alternative methods and with the goal of identifying the optimal tradeoff between adherence to in vivo conditions, quality of the results, and ease of use, the next sections will focus on key in vitro approaches (spheroids and OoC) and their application in toxicological studies.

## 3. 3D Spheroids

Spheroids represent a suitable model for improved toxicology risk assessments over other 3D cell-culture methods thanks to their faster production, higher reproducibility, and presence of cell heterogeneity within the sphere [26].

**Figure 1:**
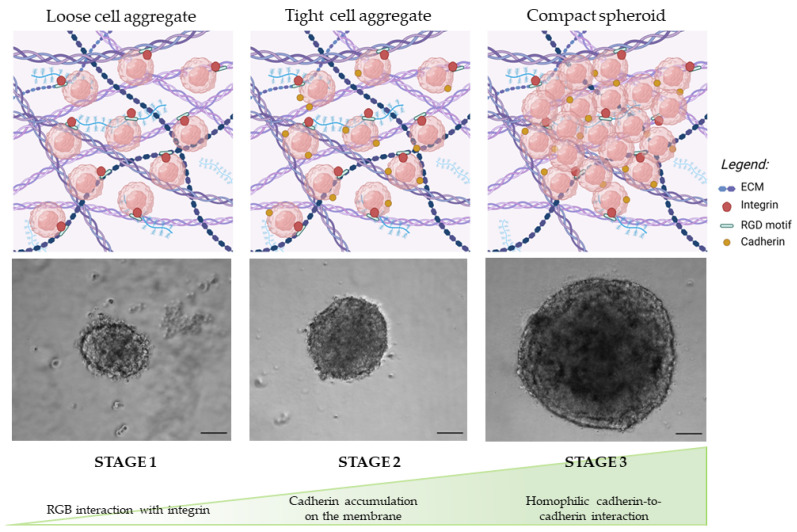
Representative optical images of SK-N-AS spheroids at different stages of growth over the course of 7 days, from the loose aggregate of cells to the formation of a compact spheroid. Scale bar = 100 μm (magnification 5×). This figure is original and unpublished.

The self assembly of a spheroid from gently pelleted cells generally follows three main steps: first, extracellular matrix arginine-glycine-aspartate (RGD) motifs bind with integrins, forming loose aggregates; second, cell–cell interactions induce increased expression of the cadherin gene, resulting in the membrane accumulation of cadherin protein; finally, a compact spheroid forms as a result of the homophilic cadherin-to-cadherin interactions [27]. Figure 1 schematizes these steps and shows representative images of the formation of a spheroid using human neuroblastoma SK-N-AS cells.

While 2D cell cultures are almost exclusively based on plastic plates or flasks, the role of both synthetic and organic materials in the formation and maintenance of spheroids is key for their optimized use. Generally, the formation methods can be divided in two main groups: scaffold-based and scaffold-free [28].

Scaffold-based methods use materials providing external cell-anchoring systems mimicking the ECM and thus supporting cell growth [29,30]. Several scaffolding biomaterials are currently available and include synthetic hydrogels, e.g., poly (ethylene glycol) (PEG), poly (lactic-co-glycolic) acid (PLGA), and polydimethylsiloxane (PDMS), and natural protein-based hydrogels, such as collagen, alginate, matrigel, gelatin, and hyaluronic acid (HA) [28,31,32]. The ideal scaffold should be chosen according to several parameters, including the porosity and the pore size (100–500 µm) [33,34], which confer specific characteristics to the 3D culture, especially in terms of the diffusion of nutrients, oxygen, and metabolites [29,35]. Natural polymers present several advantages, including biocompatibility and natural cell-adhesive properties [36]. Nevertheless, the lack of strong mechanical properties and the batch-to-batch variation makes them not easily controllable [37]. Synthetic hydrogels overcome these limitations by providing high reproducibility, stability, and control over the biochemical and mechanical properties [38]. Comprehensive reviews of scaffold-based methods can be found in the literature [29,30,39,40].

Scaffold-free methods promote the self-aggregation of cells without adding biomaterials, use specialized culture surfaces, including ultra-low attachment (ULA) plates or hanging-drop microplates [36], and exploit factors such as magnetic and gravitational forces [29]. The hanging-drop method allows the production of high numbers of spheroids, but given its high operator dependency, it leads to heterogeneous sizes and morphologies [29,41,42]. In contrast, microwell plates characterized by low-adhesion micropatterned compartments combine high-throughput production with little plate-to-plate variation [29,43]. These systems are available in different formats and an example is represented by AggreWell^TM^ plates (STEMCELL), where each of the 24 or six wells contains a matrix of pyramid-shaped microwells enabling the production of thousands of uniform cellular aggregates within 24–48 h with simple centrifugation of the cell suspensions. The exact diameter of the aggregates will be determined by the microwell dimensions and the chosen cell-seeding concentration [40]. Aggregates can be recovered from the microwells by gentle pipetting and transferred into ULA plates or bioreactors for further culturing and testing. Despite the clear advantages, this method is not ideal when analyses of single spheroids are required (such as for microscopy imaging or cytotoxicity evaluation) because of the difficulty of diluting the obtained suspension of 3D structures to the single-spheroid level.

Single spheroids can be obtained via a liquid-overlay technique or using ULA plates. The first one uses substrates creating non-adherent surfaces that favor cell aggregation [44]. Agarose diluted in medium can be used to coat the plate and prevent cell adhesion, but the generated spheroids are typically non-uniform in size and the bottom concave agarose layer may hinder some applications. Since agarose solidifies within seconds to minutes, a critical step is that it must be kept at a temperature of around 60 °C during dispension to avoid irregular or insufficient coating due to cooling. In addition, a relatively large volume (50 µL) per surface area is needed to guarantee the formation of a concave surface [45]. Finally, agarose does not interact with tumor cells, leading to its inability to activate specific pathways. Alternative materials can be used, including HA, which can interact with the surface receptors of cancer cells [44].

The ULA plates are characterized by cell-repellant surfaces promoting cell–cell interaction and self-aggregation into spheroids without the need for coating [46]. Our group has recently shown a successful application of ULA (Corning^®^, 7007, New York, NY, USA) for the formation, characterization, and evaluation of sterigmatocystin-induced cytotoxicity on neuroblastoma SH-SY5Y- and SK-N-DZ-derived spheroids [47]. The non-adhesive U-bottom surface of ULA 96-well plates allowed the formation of one centrally located spheroid per well, compatible with several applications and analyses. In particular, in the aforementioned work, the spheroids were employed for various tests, from cytotoxicity assays, such as thiazolyl blue tetrazolium bromide (MTT) and ATP assays, to immunofluorescence and Western blotting. Moreover, the round bottom of the wells directs the aggregation of cells at its center and facilitates the generation of a uniform spheroid upon centrifugation, an essential feature for the reproducibility of cytotoxicity experiments. Among the commercially available formats, we believe 96-well plates are the ideal choice for cytotoxicity experiments since they guarantee monitoring and manipulation of individual spheroids with high numbers of replicas for experimental conditions. Careful optimization of both the cell numbers and the centrifugation times needed for aggregation is necessary, as well as determination of the appropriate days of culture for spheroids to form and grow to the required size.

Dynamic culture in bioreactors brings several advantages over static methods for the generation of spheroids, including a homogeneous environment, better diffusion of oxygen and nutrients, and longer periods of cultivation [48,49]. The stirred-tank bioreactor (STR) allows an easy and large-scale production of spheroids using culture modalities that allow the control of several parameters, including oxygen and pH [48,50]. Their main disadvantage is related to the relatively high shear forces that could have a detrimental effect on the spheroids’ shape and structure. Rotating wall vessel bioreactors (RWB) are also called microgravitational bioreactors and produce spheroids in a microgravity environment with a lower shear and turbulence compared to other bioreactor systems [48,51].

### 3.1. Considerations for Spheroid Handling

To use a spheroid model for advanced toxicology risk assessment, some critical considerations need to be further addressed. First, it is crucial to have one single spheroid per well; second, the spheroids must be uniform in shape and size to reduce variability in the readouts. Although we are aware of the difficulty of obtaining an absolute standardization of spheroid culturing, we will here discuss some key points that are often overlooked.

#### 3.1.1. Morphology

Employing regular and well-rounded spheroids in in vitro assays ensures a higher reproducibility of the results [52,53]. For this reason, the evaluation of morphological parameters such as solidity and circularity is the starting step to limit the bias and choose the appropriate spheroid for a given biological application. According to Santo et al. [54], spheroids are considered regular in shape if their solidity values are higher than 0.90. Circularity (*Cir*) is used to calculate the sphericity index (SI), in turn indicating how close to a spherical geometry the construct is, according to Equation (1) [39].

(1) $$\mathrm{SI}=\sqrt{Cir}$$

**Figure 2:**
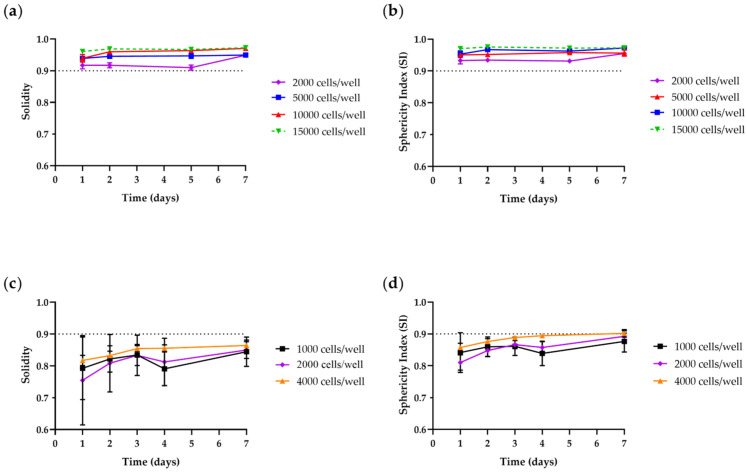
Shape parameters of BM-MSCs (**a**,**b**) and SH-SY5Y (**c**,**d**) spheroids throughout culture period in ULA 96-well round-bottom plates obtained using AnaSP software. (**a**,**c**) Solidity; (**b**,**d**) sphericity index (SI). Curves represent the mean ± SEM of 3 replicates for each cell density/well. This figure is original and unpublished.

Zanoni et al. [30] consider spheroids spherical when SI ≥ 0.90. The shape parameters may be estimated using AnaSP, a user-friendly software automatically analyzing brightfield images acquired with a standard optical microscope [55]. In our laboratories, we characterized spheroids generated from tumoral and non-tumoral cell lines. As shown in Figure 2, spheroids generated from bone-marrow-derived mesenchymal stem cells (BM-MSCs) can be considered regular and spherical in shape at all tested cell-seeding densities and culture times, with solidity values higher than 0.92 after 1 day of culture and SI ranging from 0.93 to 0.97. Regarding neuroblastoma SH-SY5Y cells, the best results were obtained for spheroids after 7 days of culture, with measured solidity values and SI over 0.85 and close to 0.90, independent of the cell-seeding concentration.

#### 3.1.2. Size and Time

**Figure 3:**
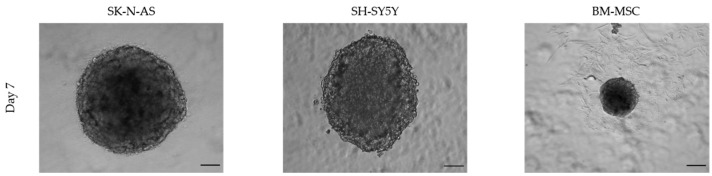
Optical images of spheroids generated from different cell lines on ULA 96-well round-bottom plates and seeding the same number of cells (2000 cells/well). Scale bar = 100 μm (magnification 5×). This figure is original and unpublished.

**Figure 4:**
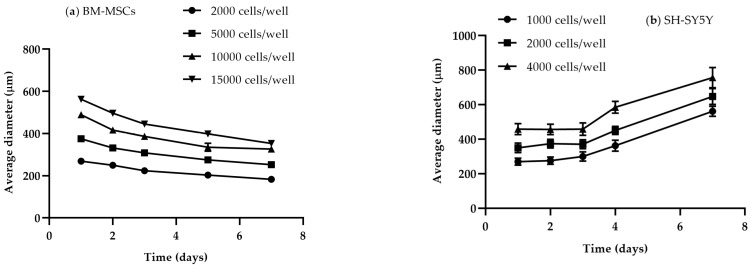
Assessments of spheroid growth on ULA 96-well round-bottom plates. Growth kinetics of (**a**) BM-MSCs and (**b**) SH-SY5Y spheroids evaluated over a period of 7 days. Curves represent the mean ± SEM of 3 replicates for each cell density/well. This figure is original and unpublished.

The physiological state of spheroids strongly depends on their size and culture time. A diameter of 400–500 µm is considered appropriate for the spontaneous formation of gradients of oxygen and nutrients and of differential proliferation rates, all essential for relevant 3D experimental studies [46]. Smaller spheroids sized up to 200 µm allow one to obtain cell-to-cell and cell-to-matrix interactions, but they do not develop a stratified composition with viable cells in the rim and necrotic/apoptotic cells in the center [55]. The seeding cell number only partially correlates to the spheroid size, which is also affected by the compactness in a cell-line-dependent manner (Figure 3). Moreover, given the specific doubling times, different cell lines require different culture lengths to obtain spheroids of a defined diameter. Therefore, the optimal seeding cell density and time in culture need to be identified for each cell line. Figure 4 shows representative size measurements of spheroids generated from BM-MSCs and SH-SY5Y cells over time in culture. While SH-SY5Y spheroids had an almost linear growth, BM-MSC spheroids showed a slight decrease, likely as a consequence of a reduction in the amount of cytoplasm and in cell volume [56].

**Figure 5:**
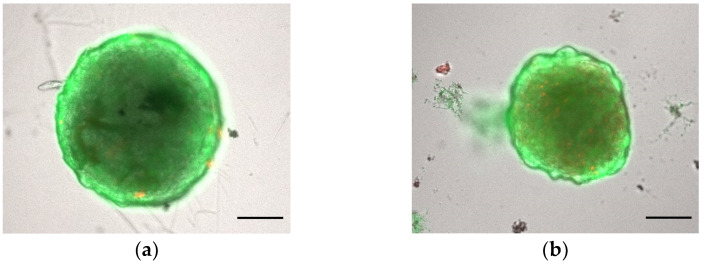
Assessments of spheroid viability on ULA 96-well round-bottom plates. Cell viability of BM-MSCs spheroids was evaluated using a Live & Dead assay after (**a**) 1 day and (**b**) 7 days of growth. Live cells are stained green and dead cells are stained red. Scale bar = 100 μm (magnification 5×). This figure is original and unpublished.

Another fundamental aspect is the assessment of the maintenance of the desired cell viability throughout the 3D constructs. Recently, several user-friendly kits have been developed to measure cell viability on 3D spheroids. Among these, the Live & Dead Viability/Cytotoxicity assay (Invitrogen^®^, L3224, Waltham, MA, USA) distinguishes live from dead cells in a population by exploiting the fluorescent properties of Calcein AM (acetomethoxycalcein) and ethidium homodimer-1 dyes (Figure 5). Calcein, as a permeable and liposoluble dye, when internalized in live cells is cleaved by intracellular esterases with the subsequent emission of green fluorescence. Ethidium homodimer-1, a cell-membrane-impermeable dye, confers red fluorescence to nucleic acids and is an indicator of cell death.

#### 3.1.3. Extracellular Matrices

**Figure 6:**
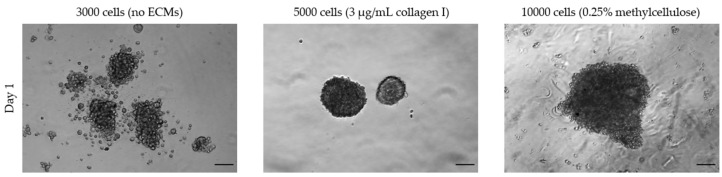
HUVEC cells seeded for spheroid formation with and without ECMs addition. Scale bar = 100 μm (magnification 5×). This figure is original and unpublished.

**Figure 7:**
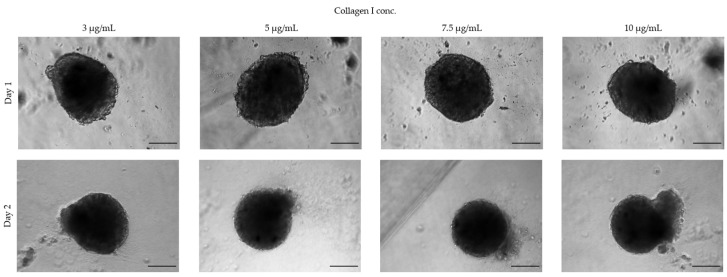
Standardizing collagen I concentration. A total of 10,000 HUVEC cells was seeded in medium supplemented with different concentrations of collagen I. Scale bar: 100 μm (magnification 5×). This figure is original and unpublished.

**Figure 8:**
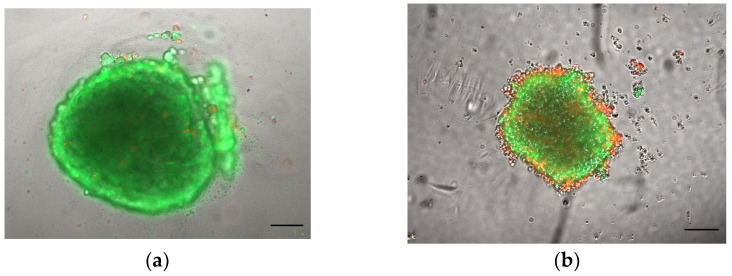
Assessments of HUVEC spheroid viability cultured in the presence of ECMs. A total of 10,000 HUVEC cells was seeded in medium supplemented with (**a**) 7.5 µg/mL collagen I and (**b**) 0.25% methylcellulose and stained with Live & Dead 1 day later. Scale bar: 100 μm (magnification 5×). This figure is original and unpublished.

Not all cell lines spontaneously form spheroids using standard culture medium but require the addition of ECMs such as collagen or methylcellulose. For example, human umbilical vein endothelial cells (HUVECs) seeded in ULA 96-well round-bottom plates form loose aggregates and only upon the addition of ECM components to the growth medium yield a 3D spheroid-like morphology (Figure 6). In this case, the spheroid-generation conditions need to be optimized by testing various ECM components. For HUVECs, we found that collagen I worked best to form spheroids with a defined boundary, with an optimized concentration of 7.5 µg/mL, while higher concentrations led to a disrupted morphology (Figure 7). Considering that some ECMs may have poor biocompatibility, their use requires a validation of their potential toxicity. The cell viability of spheroids cultured in the presence of ECMs may be tested with a Live & Dead fluorescence staining assay (Figure 8).

### 3.2. Downstream Functional Assays on 3D Spheroid Model: Advantages and Critical Issues

As previously stated, 3D spheroids improve upon the canonical 2D culture techniques by enabling us to address previously unanswered research questions [36]. Of course, bridging the gap from 2D culture techniques to 3D innovative models is not free from obstacles and challenges. In addition to the previously discussed critical aspects such as the spheroids’ formation, size, and shape, the type and method of acquisition of the viability/cytotoxicity tests following treatments with toxic substances must also be carefully considered to minimize biases and obtain reliable data.

After exposure to toxic substances, the first evidence of toxic effects is based on optical observations of potential alterations on the structural organization of the spheroids, assessed by measuring the integrity and viability of the spheroids over time [47]. Commercially available cell-viability assays can be adapted to the 3D model. An example is the CellTiter-Glo^®^ Luminescent Cell Viability Assay (Promega^®^, G968B, Milan, Italy), a luminescent assay that measures ATP to determine the number of metabolically active cells. An additional viability test applicable to 3D spheroids is the MTT assay. Compared to monolayer cultures, the MTT assay for spheroids requires slight protocol modifications. In particular, at the end of the treatment, the spheroids must be transferred to a flat-bottom 96-well plate before the MTT solution is added. However, it should be noted that the spheroid transfer could not only disturb the culture, but also lead to the loss of cell material and impede a large-scale throughput [47,57].

Moreover, a limiting step when working with 3D cultures can be the reproducibility of data. To bypass this issue and provide major robustness to the results it is mandatory to have at least four to six spheroids per experimental condition.

Microscopy and imaging are among the most widespread analytical methods employed in cell biology. However, one of the biggest issues when 3D spheroids are used as a working model is the difficulty of staining and imaging the entire structure due to the dense network of cells in the core mass. Furthermore, the plates where spheroids are formed are not always fully compatible with microscopes, hindering high-resolution imaging and often requiring spheroids to be transferred to more suitable surfaces, with consequent disturbances to the culture conditions and the potential loss of cellular material [58]. A further challenge is to capture the full complexity of the spheroid structure. To do this, a series of *xy* images can be captured at fixed steps in the vertical direction using automated microscopes to obtain a *z* stack [58]. Moreover, since not all antibodies labeled with fluorescent dyes are able to diffuse into and stain the inner core of the spheroids, we recommend performing primary and secondary antibody incubations under constant and gentle shaking to improve upon the limited penetration capacity.

Spheroids are also adaptable to routine molecular and biochemistry applications. A recent study optimized the protocol to determine the protein profile in 3D cultures after mycotoxin treatments [47]. Surely, one aspect to consider for specific downstream applications is the overall number of viable cells available at the desired endpoint. A simple but not necessarily easy strategy is culturing high numbers of spheroids per condition to obtain the quantity and purity of RNA and proteins suitable for the analyses. After the careful collection of spheroids, the genes and proteins of interest can be detected according to standard PCR and blotting procedures.

Other assays that have been shown to be adaptable to the spheroid model are cell cycle and apoptosis analysis using flow cytometry. The crucial aspect to consider for this type of acquisition is the sensitivity of the cytometer, with a minimum cell count requirement of 500,000 cells, thus requiring large numbers of spheroids according to the initial seeding density, growth timing, and final cell number obtained [47]. The experimental procedure is based on the labeling and acquisition of single-cell suspensions; thus, a defined number of spheroids per experimental condition must be collected and trypsinized before staining and subsequent cytometer acquisition [59].

Angiogenesis is a multi-step process involving the parent vessel’s extracellular matrix degradation, causing the migration and proliferation of endothelial cells to form a new vessel with a lumen and a layer of mural cells [60]. Being a complex phenomenon, reproducing it in vitro is a challenge not addressable with standard cell culture. Dr. Thomas Korff and Dr. Hellmut Augustin pioneered an endothelial cell spheroid-based 3D angiogenesis technique for in vitro studies [61]. Based on this system, in vitro angiogenesis assays were widely developed to investigate the putative angiogenesis-related toxic effects of compounds and/or genetic manipulations [62]. By culturing endothelial-cell spheroids with the hanging-drop method and embedding them into a collagen matrix, the ability to form capillary-like tubes can be investigated by counting the number and length of sprouts after treatment with toxins [63]. The spheroid-based sprouting assay is an advantageous tool that makes the study of angiogenesis in an in vivo-like microenvironment easier and more robust. Similarly, 3D cultures can be instrumental in analyzing with better soundness the general cell-migration phenomenon. For this purpose, different matrices have been tested to include the spheroids, such as gelatin, collagen, and matrigel [64,65,66]. Based on our experience, a gelatin coat guarantees a good experimental reproducibility and spheroids generated in ULA 96-well round-bottom plates can be transferred to gelatin-coated “migration” plates and monitored over time using an optical microscope. Within a few hours, tumor cells begin spreading from the spheroid over the coated surface [47]. Fundamental for correct data interpretation are the image-processing steps performed on several images for each sample. The complete image-analysis routine needs to be managed with different and sequential image-processing techniques with the aim of measuring the cells spreading on the gelatin coat. The Sobel mathematical model, based on the edge detection of optical greyscale images, provides a reliable measure of migrating cells. The edge of the spheroid masks detected at time 0 is employed to identify the inner regions from the surrounding migrated cells [47,67,68].

**Figure 9:**
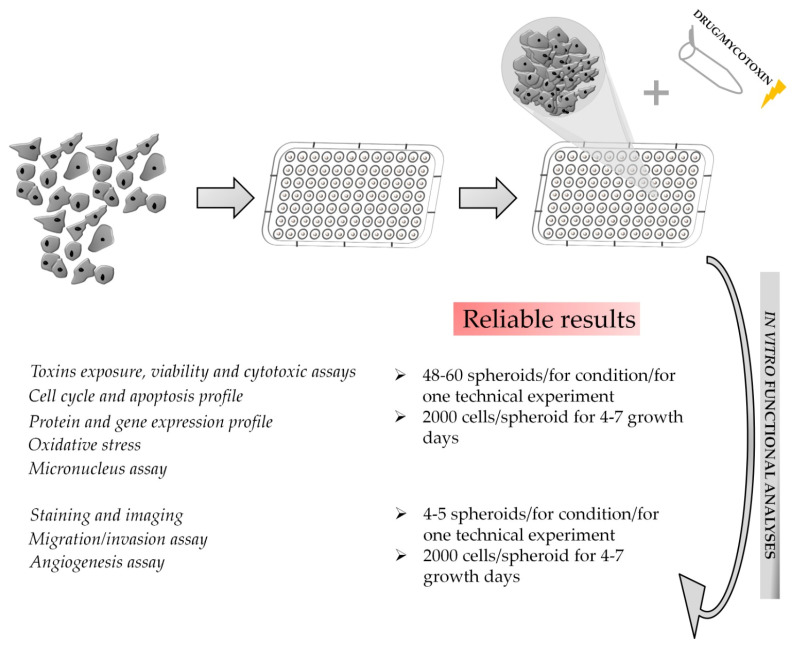
Summary of functional assays and possible approaches for the obtainment of reliable results in a spheroid model. This figure is original and unpublished.

Regarding the assessment of genotoxicity associated with exposure to toxic substances, there are many limitations associated with 2D cultures, as extensively reviewed previously [69]. Moreover, the regulated genotoxicity in vitro testing methods have demonstrated low specificity and produced misleading false positives, resulting in the need for additional animal testing [70]. Recently, 3D in vitro models have had increasing application in the field of genotoxic assessment due to their reliability in reproducing the physiological environment and metabolism of chemicals [71]. An existing application tested genotoxic effects on 3D HepG2 cells and compared the effects in 2D monolayer cultures, showing that the spheroids had improved sensitivity in detecting genotoxic compounds evaluated with a comet assay [72]. Genotoxicity assessments, like other analyses described above, require at least 48–60 spheroids per condition to provide reliable results (Figure 9). Other advanced tools are the 3D skin comet and the reconstructed skin micronucleus (RSMN) assays [73], which detect DNA lesions and chromosomal damage, respectively, that could be due to exposure to a variety of compounds found in cosmetics, industrial chemicals, and household products. In the field of dermally applied chemical exposure, the 3D skin comet assay resulted in a 70–100% range of predictivity and good intra- and inter-laboratory reproducibility.

In addition, novel genotoxicity testing enabled researchers to observe DNA damage through a high-throughput comet chip assay on metabolically competent HepaRG cells [74]. The optimized 3D culture system used 96- or 384-well ULA plates for evaluating the response to various direct-acting and indirect-acting genotoxicant/carcinogen exposure. The high-throughput comet chip platform on 3D spheroids proved to be a reliable in vitro approach improving the risk assessment for human genotoxic carcinogen exposure.

Another common effect associated with toxin exposure is the induction of oxidative stress. Currently, the role of reactive oxygen species (ROS) on decreased cell viability induced by toxic substances is mainly investigated using simplified 2D monolayers [72]. The use of a 3D spheroid model enables the capture of the complexity of these biological processes and cellular behaviors, better resembling in vivo conditions. ROS generation can be measured using an ROS-specific fluorescent dye such as 2’,7’-dichlorodihydrofluorescein diacetate (DCFH-DA), a cell-permeable probe used to detect intracellular ROS through plate-reader acquisition [47]. In a 3D glioblastoma model, the CellROX Green Reagent (Thermo Fisher Scientific, Waltham, MA, USA) was used to measure the release of intracellular ROS. CellROX, a DNA-binding cell-permeant dye, displays green photostable fluorescence if oxidation is active. This fluorescence followed the fluorescent probe’s incorporation due to drug treatments. To provide further insights on the oxidative status of the spheroids, the authors also assessed glutathione (GSH) levels using a luminescence-based assay (Promega, Madison, WI, USA) as well as the expression of glutathione peroxidase4 (GXP4) and 8-Oxo-2′-deoxyguanosine (8-oxo-dG) using immunohistochemistry in sections from the spheroid tumors [75]. The expression of enzymes associated with ROS metabolism can also be measured via PCR and/or Western blot. These time-consuming methods require a skilled operator and, to reduce the data variability, multiple technical replicates per condition [47].

## 4. Organ-on-a-Chip (OoC) Systems

OoCs combine cell biology and microfluidic technology in a microdevice capable of recapitulating the dynamics, functionality, and (patho)physiology of human organs. Typically, a microfluidic-based device houses cell constructs embedded in engineered 3D microenvironments, with medium flow driven by pumps (syringe or peristaltic). Compared to conventional 2D cultures, cells in OoC systems show a cell polarization and cytoskeletal architecture more similar to those observed in vivo, in addition to maintaining cell viability and functionality for longer periods of time. OoCs also enable a fine regulation of the biological and physicochemical micro- or nano-environment thanks to a tight control of the flow of the culture medium. The flowing medium mimics the continuous supply of oxygen and nutrients, mirroring the in vivo physiological conditions while also allowing the delivery of drugs or other compounds of interest to the cells in culture. Another advantage derives from the use of small volumes of culture medium and test substances, with great economic gain that should not be underestimated.

So far, OoCs have greatly advanced life science and medical research, playing an increasingly important role in preclinical trials and drug development, but also provide new broad opportunities for the toxicological field. With the ability to recapitulate in vivo physiology and study complex biological systems in a controlled in vitro environment, OoCs predict more accurately the acute and chronic effects induced by chemical and natural toxicants to which humans may be exposed. These advanced cell-culturing systems promote the improvement of toxicological hazard and risk assessment and overcome ethical limitations such as the use of animal testing for toxin risk assessment.

Although OoCs could play an important role in the revolution of toxicology, their application shows downsides, such as the low throughput, difficulty of standardization and validation, and most importantly, the inability to give ultimate answers on the adverse systemic effects at the level of the whole individual. The following sections provide an overview of the usability, compatibility, and assay ability aspects of OoC systems.

### 4.1. Considerations for OoC Handling

In OoC technology, the design, fabrication methods, and culture strategy strongly depend on the ultimate experimental purposes. All the choices relating to the development of an OoC model must depend on them, from the selection of materials to the cell source.

#### 4.1.1. Material Selection

There are various materials that can be used for the fabrication of OoCs; however, each of them shows advantages and disadvantages. Here, we present only the most important and widely used materials in OoC design and development.

The most commonly used material is PDMS, a silicone polymer whose biocompatibility, elasticity, optical transparency, and gas permeability make it the ideal candidate for biological experiments [76]. However, its tendency to adsorb a wide range of chemicals limits its application in toxicity studies and must be carefully taken into account [77,78]. The absorption of biomolecules is reduced when using glass, whose great optical transparency also makes it the first choice for real-time imaging. However, glass chips are not suitable for long-term cell culture, since they are not gas-permeable [79]. Furthermore, in contrast to PDMS, which can be processed with soft lithography and micromolding techniques, glass is typically processed with the more expensive and time-consuming standard lithography [76,80].

Recently, plastic materials such as poly(methylmethacrylate) (PMMA), polycarbonate (PC), and polystyrene (PS) have been increasingly replacing the traditional PDMS- and glass-based chips due to their low cost and easy fabrication [81]. Furthermore, they show several interesting properties, such as: (i) high biocompatibility, enabling cell growth and adhesion; (ii) excellent optical transmittance, allowing high-quality fluorescent imaging; and (iii) resistance to small-molecule permeation and, therefore, approval by the Food and Drug Administration (FDA) [82,83]. Nevertheless, there are some limitations in their use, due to the low gas permeability, which has a negative impact on long-term experiments, and their incompatibility with most organic solvents [84,85].

Hydrogels are polymeric materials with a high water content showing many attractive properties for the OoC field, including biocompatibility, high permeability, biodegradability, elasticity, and low cytotoxicity [37]. In addition, their mechanical properties mimic some elements of ECMs, also protecting biological entities in long-term studies [86]. Due to their high biocompatibility and presence of cell-binding sites, hydrogels are mostly coated on the surface of OoC devices fabricated with a material with less-favorable cell-attachment properties [87,88]. However, hydrogels, especially natural ones, present some limitations due to the poor stability and batch-to-batch variability. Collagen is one of the most widely used hydrogels for bioengineered tissues, being the most common ECM component in the human body [89]. Techniques for hydrogel-based OoC fabrication include lithography, 3D printing, and molding [90].

Finally, as for conventional cell-culture platforms, all components of the OoC devices must be sterile, and the choice of sterilization method depends on the materials involved. An inappropriate sterilization may result in component damage, ultimately leading to system failure. The conventional autoclave sterilization methods cannot be used for materials with low thermal resistance, such as PMMA and PC plastics, and should be replaced with UV and/or ethanol treatments. However, materials that exhibit opacity and/or the ability to absorb UV rays are not suitable for UV sterilization, while ethanol is not compatible with materials that can absorb it, including PDMS [91].

Once sterilized, the device surfaces that will be in direct contact with cell cultures may require specific treatments to ensure biocompatibility and regulate cell attachment. If adhesion needs to be favored, ECM coating is often adopted (see above), while pluronic acid treatment, which prevents cell adhesion, can be used in those chips designed for 3D culture [3].

#### 4.1.2. Selection of Cell Culture

Closely related to the scientific question is the selection of the appropriate cell source. Immortalized cells, primary cell cultures, and induced pluripotent stem cells (iPSCs) can be used. Cancer cells are widely employed in toxicological studies because they are highly proliferative, easy to culture, and well-characterized. Despite these advantages, immortalized cell lines show an altered genotype and phenotype, which limit their ability to reproduce physiological cell behaviors [92]. For these reasons, primary cells are a valid option to evaluate the toxicological effects induced by exposure to toxic substances under physiological conditions. However, they show several limitations, including poor accessibility due to the lack of donors, inter-donor variability, limited proliferative capacity, and the loss of tissue-specific functions when maintained in vitro, preventing their use in chronic toxicity studies [93,94].

The use of iPSCs is in demand in toxicological studies using OoC models. The main advantages of iPSCs are their capacity to differentiate in various cell lineages [95] and their adult origin, avoiding the ethical concerns associated with the use of embryonic tissues. In addition, like primary cells, iPSCs derived from donors with known disease phenotypes inherit the patient genotype, making them ideal to study toxic responses for susceptible groups, but less suitable for broader populations [95]. The major challenge in the use of iPSCs is their correct differentiation into specific cells or tissues. Due to their natural variability, standardization remains difficult, but protocols to maintain, differentiate, and mature iPSCs in vitro are constantly being developed and updated. In addition, direct on-chip culture techniques have also been performed using iPSC-derived intestinal organoids [96], proving that a synergy between iPSC culture and the OoC technology is expected to greatly progress research.

OoCs culturing monolayers of only one cell phenotype might underrepresent the in vivo complexity. For these reasons, new approaches include more complex 3D architectures with multicellular compartments [97]. When culture wells allow direct access (open at the top), 3D cultures previously formed with scaffold-free techniques can be directly seeded without the need for delicate injections through microchannels [98,99,100,101].

Single-organ systems are ideal to study the effects of toxicants on a specific target; however, a more comprehensive analysis would require a multi-organ system that would reproduce the correlations that one site has on the functionality of another one. However, obtaining true multi-organ systems is not as straightforward as the simple connection of two or more single-organ systems. For example, multi-OoCs require an optimized formulation of a common culture medium, capable of ensuring and maintaining the correct viability and physiology of each cell population. Mixing the culture media used for each cell type has been shown to generally produce good outcomes [102,103]. However, as the number of cell types in a system increases, optimizing a co-culture medium can become more challenging. In these cases, the compartmentalization of cells into semi-insulated culture chambers can be a viable option to circumvent the issue [99,104]. 

### 4.2. Downstream Function Assays on OoC Model: Advantages and Critical Issues

The functional assays that can be performed in OoC systems can generally be divided into two classes: on-chip and off-chip. The first comprises immunohistochemistry, trans-epithelial electric resistance (TEER), and migration and angiogenesis assays. Off-chip assays include high-performance liquid chromatography/mass spectrometry (HPLC/MS), gas chromatography/mass spectrometry (GC/MS), and enzyme-linked immunosorbent assays (ELISA) [105].

Immunohistochemical staining and other microscopy readouts are the most widespread methods used in OoC devices. By using the microchannel network of the OoC, it is possible to directly deliver the fluorescent stains or antibodies to assess cell viability or the expression of specific markers. Furthermore, the use of optically transparent materials ensures excellent imaging quality [106,107,108,109] while also enable on-line monitoring of cell behavior (if live cell stains are available, i.e., in reporter cell lines). Other on-chip assays (e.g., calcium-imaging, colorimetric, and luminescence assays) are still feasible but less frequently adopted due to the difficult optimization in a microfluidic environment.

The measurement of TEER values allows the evaluation of the integrity and permeability of any barrier tissue. This technique is mainly employed in OoCs where cells form a natural barrier between fluid compartments. TEER is easily measured by applying Ohm’s law, but impedence spectoscopy, using microelectrodes integrated into the chip during fabrication [110,111] or before an experiment [112,113], provides a more accurate measure. Complex systems containing several cell-culture chambers, such as the one reported by Ramadan et al. [114], which measured the barrier integrity of human keratinocytes in co-culture with monocytes, require highly trained end-users.

Off-chip assays have the advantage of measuring many markers simultaneously. However, only approaches that require a few microliters of sample volume are compatible with microfluidic chips. Perhaps for these reasons, HPLC/MS and GC/MS are used by few in OoC studies [115,116,117]. To meet the needs of OoC systems, in recent years various microarray techniques and commercial assay kits have been developed for specific biomarkers using very low volumes (<5 µL) [91].

Due to the high sensitivity of HPLC/MS and GC/MS techniques and their ability to provide quantitative analysis, considerable efforts have been made by researchers to overcome the difficulties encountered in coupling OoC to off-chip mass spectrometers. However, with the continued development of microfabrication technology, coupling of microfluidic systems to MS has become more common [118]. It must be noted that producing a device with chromatographic separation for LC/MS systems remains challenging due to the high back pressure generated when pushing the mobile phases through a particle-packed channel. Recently, Chen et al. developed a reproducible, robust, and stable 3D bioprinting microfluid chip coupled to LC/MS, demonstrating the suitability of the device for drug analysis and straightforward quantification [119]. The field of development of new coupling strategies is still growing and oriented towards the use of miniaturized analyzers, which would greatly increase the possibility of on-site analysis [120].

Lytic, transcriptomic, proteomic, and cell-viability assays typically require the retrieval of the cell samples from the chip prior to analysis, thus increasing the operator dependency. It is also essential that the chip be designed to enable sample retrieval without detrimental effects on their function and structure in order not to affect the results [121].

It should be noted that most publications reported performing the analysis only on a single chip, although it is well-accepted that a minimal number of replicas per condition as well as positive and negative controls is required to produce statistically relevant and reliable data.

## 5. Applications of Advanced In Vitro Models for Mycotoxin Assessment

There is promising evidence that the toxicology field could benefit from the application of alternative culture methods. An innovative approach could contribute to a more reliable toxicity evaluation of compounds and support regulatory agency policies on allowable exposure levels. To date, regulatory toxicology has only partially embraced alternative methods, and food toxicology in particular is a sector still largely outside of the 3R and Tox-21c proposed regulations [122]. Searching terms as “food” AND “toxicology” AND “alternative methods” in PubMed results in a bibliography of just 118 articles from 1985 to the present. Of these, very few address mycotoxins and their risk assessment. On the contrary, searching terms such as “mycotoxin” and “animal model” yield 1200 articles in the last 20 years only, highlighting how far this topic is from adopting alternative approaches.

Mycotoxins are toxic secondary metabolites produced by filamentous fungi frequently found as contaminants of food and feed. Recently, Royal DSM, a global science-based company in nutrition, health and sustainable living, released Biomin results for the 2020 World Mycotoxin Survey, identifying 65% of analyzed samples contaminated with at least one mycotoxin above the threshold levels [123]. Mycotoxins are objects of concern, as they are known to induce adverse health effects in humans and animals following the consumption of contaminated foodstuffs. To prevent and contain the negative effects on consumers and animals, regulatory limits and guidance values are stipulated in several countries for many mycotoxins [124]. So far, mycotoxin risk assessment mainly relies on studies evaluating their cytotoxicity using in vitro 2D cell models, which can overestimate or underestimate the cellular toxicity due to the lack of a 3D architecture. In fact, evidence is accumulating that the same degree of sensitivity of the 2D systems did not translate into 3D culture systems [57,125,126]. As described in the sections above, this discrepancy can be attributed to the presence of more pronounced intracellular junctions in 3D cell models, mimicking physiological barriers, as well as a dense ECM, which influences xenobiotic transport [127,128]. In agreement with the literature, 3D models exposed to the mycotoxins ochratoxins (OTs), citrinin (CIT), sterigmatocystin (STE), and fumonisin B1 (FB1) showed a remarkably different sensitivity compared to monolayer cells [47,129,130]. These data shed light on the need for a more precise evaluation of mycotoxin risk using more complex models.

A list of studies aimed at assessing mycotoxin toxicity using alternative methods is provided in Table 1. Aflatoxin B1 (AFB1) is the mycotoxin that is most frequently studied using alternative in vitro models. The already-known genotoxic potential of AFB1 has recently been further investigated using hepatic spheroids, which show a higher expression of metabolic enzymes than 2D monoculture and, thus, better represent the metabolic activity of the hepatic cells in vivo [131,132]. Likely because of the increased metabolic activity of the spheroids, 3D HepG2 spheroids demonstrated a greater efficacy than standard monolayer cells in detecting genotoxicity, showing a higher sensitivity to low concentrations of AFB1 [71,72]. These data confirm that the advanced features of the 3D model make it an ideal candidate to improve the state-of-the-art for AFB1 genotoxicological assessment. The acute toxicity of AFB1 was also assessed on a microphysiological system (MPS) that enabled the maintenance of the hepatic functionality of hepatocytes for at least 14 days. An analysis of the LDH activity showed a modest increase in LDH after exposure to 30 µM AFB1. The system has proven to be a valuable tool for evaluating the hepatotoxicity of toxicants that require bioactivation, such as AFB1. However, only one liver cell type was employed in the system, representing a limitation of the approach [106]. Based on the study by Ma et al., an even more reliable toxicity evaluation of AFB1 may be obtained using 3D co-culture spheroids. In fact, the authors observed an important difference in terms of metabolic viability and sensitivity of 3D co-culture spheroids compared not only to 2D cells, but also to 3D mono-type cell spheroids [133]. Specifically, AFB1 (≤30 µg/mL) was shown to induce apoptosis in 2D HepG2 cells, but did not significantly affect 3D cell spheroids, especially those in triple co-culture, which exhibited a higher resistance. Combined with an analysis of gene expression, both metabolic activation and detoxification efficiency were higher in 3D than in 2D cells, explaining the different sensibility shown by the two models. Similarly, in another study, the authors observed different interactions associated with the co-exposure of AFB1 with cyclopiazonic acid (CPA) compared with those reported in the literature. Synergistic effects were obtained in co-cultured hepatocyte spheroids at concentrations close to real-life exposure levels (0.625 µg/mL AFB1 and 3.125 µg/mL CPA) [134], and the authors speculated that this could be because the 3D culture environment might confer metabolic processes that differ from those of adherent cells, leading to differences in interactions between 3D and 2D cells. Since it is widely accepted that the carcinogenic and toxic effects of AFB1 are dependent on its metabolic activation [135], 3D cell culture seems to be a complex and suitable model for a more precise evaluation of mycotoxin co-exposure.

Considering that the toxicity of mycotoxins can be influenced by other organs once in the bloodstream, Bovard et al. developed a microfluidic multi-organ chip that overcomes the inaccuracies associated with 3D models cultured under conventional static conditions [136]. The novel system consisted of a 3D organotypic bronchial model connected with liver spheroids acting as a metabolizing compartment. In normal human bronchial epithelial cells at the air–liquid interface, AFB1 cytotoxicity was delayed by co-culture with liver spheroids, suggesting that at least part of AFB1 was metabolized into the less-toxic parent compound aflatoxin Q1 (AFQ1) by the hepatic compartment. Similarly, Schimek et al. observed that AFB1 induced a greater decrease in functionality and viability in mono-cultured bronchial cultures compared with lung–liver co-cultures [137]. Taken together, these studies lay the first stone upon a new alternative and physiologically relevant approach to assess the potential toxicity of AFB1.

For many mycotoxins of concern, the mechanisms underlying their toxicity are still elusive due to the lack of adequate models that fully recapitulate human functions in vivo. Imaoka et al. used a 3D human kidney proximal tube microphysiological system (kidney MPS) to define the dose–response relationships of ochratoxin A (OTA)-induced nephropathy [138]. The LD_50_ values obtained by the authors (1.21 and 0.375 µM at 72 and 186 h of exposure, respectively) agreed with the clinically relevant toxic concentrations of OTA in urine (0.37 µM), suggesting that the kidney MPS may represent a good model to reflect chronic toxicity of OTA and support changes in its risk assessment.

For the mycotoxin deoxynivalenol (DON), a deeper understanding of its effects on intestinal barrier functions is needed, considering that it is rapidly and almost completely absorbed in the proximal small intestine. However, the effects of DON on intestinal stem cells have not yet been studied in vitro due to the lack of a model containing them. To overcome this issue, Hanyu et al. used intestinal organoids containing intestinal stem cells (enteroids) to evaluate DON toxicity on luminal and basolateral intestinal sides [139]. Enteroids consist of a central lumen lined by a villus-like epithelium and several crypt-like domains and allow mimicry of most features of the native intestine. In the study, DON was delivered to the enteroid lumen using a microinjection technique and its basolateral exposure was shown to affect intestinal stem cells more than luminal exposure at equal concentrations. Similar results were obtained using the native small intestine of mice exposed to DON orally. DON toxicity in enteroids was also reported by Li et al., who showed that acute exposure to DON suppresses intestinal-stem-cell-based enteroids’ expansion [140]. These findings support the use of enteroids as a powerful alternative tool to test the effects of toxins on intestinal stem cells. Very recently, DON-mediated effects on intestinal barrier leakage were further confirmed using a three-layered 3D gut-on-a-chip Caco-2 cell-culture model that allowed the inclusion of intestinal flow, ECM, and compartmentalization in three compartments (apical gut tube with lumen, ECM, and basolateral compartment). Similar to the previous findings, the intestinal cells were found to be more sensitive to basolateral DON exposure [141]. Noteworthy, with the addition of the third dimension and the application of fluid flow, shear stress, and the integration of the ECM, this study took one step further towards a more physiological model solution.

**Table 1:** Studies assessing mycotoxin toxicity using alternative in vitro methods.

Mycotoxin	3D Model	Endpoint	Reference
AFB1	Hepatic spheroids (HepG2 cells)	DNA damage	[72]
	Hepatic spheroids (HepG2 and HepaRG cells)	Cytotoxicity, liver functionality, genotoxicity	[71]
	Human hepatocytes cultured in a MPS	LDH release	[106]
	Mono-type spheroids (HepG2 cells); co-cultured spheroids (HepG2 cells + EA.hy 926 cells); triple co-cultured spheroids (HepG2 cells + EA.hy 926 cells + LX-2 cells)	Cell viability, mitochondria, oxidative stress, cell membrane	[133]
	Normal human bronchial epithelial (NHBE) cells cultured at the air–liquid interface (ALI); lung/liver-on-a-chip (NHBE ALI + HepaRG spheroids)	Transepithelial electrical resistance (TEER), ATP content	[136]
	Lung/liver-on-a-chip (Bronchial MucilAir + HepaRG and HHSteCs spheroids)	Intracellular ATP levels, LDH release	[137]
	Paper-based 3D HepG2 culture	Hepatotoxicity at different oxygen tensions	[142]
AFB1, CPA	Triple co-cultured spheroids (HepG2 cells + EA.hy 926 cells + LX-2 cells)	Individual and combined cell viability, mitochondria, oxidative stress, metabolomic analysis	[134]
STE	Human neuroblastoma spheroids (SH-SY5Y and SK-N-DZ cells)	Cell viability, oxidative stress, apoptosis, DNA damage, migration	[47]
CIT, OTs,	Canine kidney spheroids (MDCK cells)	Individual and combined cytotoxicity	[129]
DON	Mouse enteroids	Intestinal barrier function	[139]
	Porcine enteroids	Intestinal stem cells activity	[140]
	3-layered 3D gut-on-a-chip Caco-2 cell culture	Intestinal barrier function	[141]
FB1	Rat hepatic spheroids	Cytotoxicity	[130]
	3D human esophageal epithelial cells (HEEC)	Cell viability	[143]
OTA	3D human kidney proximal tubule microphysiological system	Cytotoxicity, analysis of kidney injury biomarkers, OTA transport, detoxification, and bioactivation	[138]

AFB1: Aflatoxin B1, ALI: air–liquid interface, CIT: citrinin, CPA: cyclopiazonic acid, DON: deoxynivalenol, EA.hy 926: immortalized human vascular endothelial cells, FB1: fumonisin B1, HHSteCs: human hepatic dtellate cells, LDH: lactate dehydrogenase, LX-2: human hepatic stellate cell line, MPS: microphysiological system, NHBE: normal human bronchial epithelial, OTs: ochratoxins, OTA ochratoxin A, SK-N-DZ: human neuroblastoma MYCN-amplified cells, STE: sterigmatocystin.

## 6. Conclusions

Both 2D and 3D cell-culture methods allow us to obtain an advanced understanding of the cellular response to toxin exposure. However, 3D models have proven to have the potential to better recapitulate the in vivo architecture of natural tissues and organs. Thus, researchers working to test toxins, including mycotoxins, on 2D cell monolayers should seriously consider 3D cell-culturing alternatives. Although there is encouraging evidence that the toxicological field could benefit from the application of advanced and complex culture methods, it has only partially embraced them, especially in the field of regulatory toxicology. Several alternative methods for assessing toxicity endpoints, such as irritation, corrosion, and genotoxicity, have already been validated and accepted by the Organization for Economic Co-operation and Development (OECD) [144,145]; however, there is still a lack of alternative models for many other toxicity endpoints. One of the reasons for the slow adaptation to alternative methods could be due to the difficulties of standardization, quality control, and validation. Furthermore, some alternative methods, while representing a successful example of in vitro reproduction of the in vivo microenvironment, are technically challenging and lack in throughput. Indeed, as the complexity of the system increases, the design, fabrication, and other phenomena commonly ignored in conventional macroscale cell cultures, such as bubble formation, evaporation, and nutrient depletion, assume a key and fundamental role. One of the major benefits of alternative in vitro models is the great potential to increase the biological relevance of in vitro studies. The development of cheaper and more user-friendly alternative systems would facilitate the adoption of this technology by more laboratories. With a shift in methodology and an increased effort for alternative models, the techniques will be better understood, and more advanced methods will be developed and adopted for toxicology studies.

## Data Availability

Not applicable.
